# Nitric Oxide Signaling and Neural Stem Cell Differentiation in Peripheral Nerve Regeneration

**Published:** 2010-06-14

**Authors:** Jessica Tao Li, Chandra Somasundaram, Ka Bian, Weijun Xiong, Faiz Mahmooduddin, Rahul K. Nath, Ferid Murad

**Affiliations:** ^a^Brown Foundation, Institute of Molecular Medicine, Houston, TX 77030; ^b^Texas Nerve & Paralysis Institute, 6400 Fannin St, Houston, TX 77030

## Abstract

**Objective:** The objective was to examine whether nitric oxide signaling plays a role in human embryonic stem cell differentiation into neural cells. This article reviews current literature on nitric oxide signaling and neural stem cell differentiation for potential therapeutic application to peripheral nerve regeneration. **Methods:** Human embryonic H9-stem cells were grown, maintained on mitomycin C–treated mouse embryonic fibroblast feeder layer, cultured on Matrigel to be feeder-free, and used for all the experiments. Fluorescent dual-immunolabeling and confocal image analysis were used to detect the presence of the neural precursor cell markers nestin and nitric oxide synthase-1. Fluorescence-activated cell sorting analysis was used to determine the percentage of expression. **Results:** We have shown the confocal image of stage 1 human embryonic stem cells coexpressing nestin and nitric oxide synthase-1. Fluorescence-activated cell sorting analysis indicated 24.3% positive labeling of nitric oxide synthase-1. Adding retinoic acid (10^−6^ M) to the culture medium increased the percent of nitric oxide synthase-1 positive cells to 33.9%. Combining retinoic acid (10^−6^ M) with 8-brom cyclic guanosine monophosphate (10^−5^ M), the fluorescence-activated cell sorting analysis demonstrated a further increase of nitric oxide synthase-1 positive cells to 45.4%. Our current results demonstrate a prodifferentiation potency of nitric oxide synthase-1, stimulated by retinoic acid with and without cyclic guanosine monophosphate. **Conclusion:** We demonstrated for the first time how nitric oxide/cyclic guanosine monophosphate signaling contributes to the development of neural precursors derived from human embryonic stem cells and enhances the differentiation of precursors toward functional neurons for peripheral nerve regeneration.

Financial support was provided by the John S. Dunn (74Y-1-9135 and 000447) and Robert A. Welch Foundations (AU-1437 and L-AU-0002); National Institute of General Medical Sciences grant (GM-076695-02); the Department of Defense (T5-0004271 project); as well as the University of Texas and the Shanghai University of TCM. We also acknowledge supports from the E-Institutes for Nitric Oxide and Inflammatory Medicine (E-04010); Key Project (2006BAI11B08-03) from the Ministry of Science and Technology; and projects 08430711300 and 08DZ1972104 of Shanghai Committee of Science & Technology. This work was also supported in part by Texas Nerve and Paralysis Institute.

## PERIPHERAL NERVE INJURY

Peripheral nerve injury is a common and devastating clinical problem. Traumatic events due to accidents or violence cause peripheral nerve damage in 2.8% of these patients.^1^ Obstetric nerve injury due to breech delivery affects 0.38 to 2.6 out of every 1000 births,^2^ leading to considerable long-term disability.^3^ Traumatic nerve injuries are often associated with life-threatening injuries, which must be treated primarily with great care. Injury to peripheral nerves may result in demyelination or axonal degeneration and, eventually, loss of motor and/or sensory function.^1^,^4^^-^7 Recovery of function occurs with remyelination, axonal regeneration, and reinnervation of the sensory receptors and/or muscle end plates.^7^,^8^ The relatively slow rate of peripheral nerve regeneration presents a challenge to the recovery of nerve function.^9^ Complete recovery is fairly infrequent, misdirected, or associated with debilitating neuropathic pain.^5^ Satisfactory results occur following relatively minor injuries such as neurapraxia or axonotmesis.^10^,^11^ The outcome remains relatively poor following severe (fifth degree) nerve injury, or neurotmesis,^10^,^11^ which describes a rupture or avulsion of the nerve.^12^ The extent of the injury has been classified and described by Seddon,^10^ Sunderland,^12^ and Mackinnon and Dellon.^13^ Effective treatments of degenerative and traumatic diseases of the peripheral nervous system are not currently available.

## ALTERNATIVE REPAIR STRATEGIES

The use, advantages, and disadvantages of both autologous nerve grafts (autografts) and synthetic nerve guide conduits in nerve repair strategies have been discussed in detail.^14^^-^17 Although autografts offer the best results in nerve reconstruction, their disadvantages include donor site morbidity, sacrifice of a functional nerve, formation of potential painful neuromas, structural differences between donor and recipient grafts, and insufficient graft material.^18^,^19^ The advantages of artificial nerve guide conduits include their availability and ease of fabrication. However, clinical outcomes associated with the use of artificial nerve conduits are often inferior to that of autografts, particularly over long lesion gaps.^15^ The disadvantages of both methods have been described.^17^

In 2009 and 2010, Radtke et al^20^^-^23 have described a number of cells derived from adult peripheral tissues, including Schwann cells (SCs) from peripheral nerve, olfactory ensheathing cells (OECs), and adipose tissue–derived mesenchymal stem cells (MSCs), all being actively investigated for cell therapies targeting peripheral nerve regeneration. They have demonstrated that transplanting peripheral myelin–forming cells (SCs and OECs) into the site of microsurgical nerve repair leads to improved regeneration and functional outcome by providing a structural scaffold for regeneration and secreting neurotrophic factors such as nerve growth factor.^23^ Committed cells such as OECs and SCs that are manipulated minimally and expanded in culture may pose less risk of tumorogenecity but have the disadvantage of limited cell harvest yields. In selecting cells for therapeutic intervention of peripheral nerve repair, this balance between in vitro transformation of expanded cell lines and the limitation of cell harvest yields must be considered.

Walsh and Midha^17^ summarized the work of several studies that focused on the use of stem and precursor cells from different sources (bone marrow,^24^,^25^ skin,^17^,^26^,^27^ hair follicles,^28^,^29^ adipose tissue,^30^,^31^ human amniotic fluid MSC,^32^ and embryos^33^,^34^) as alternative and adjunct candidates to repair injured nerve. The transplanted stem cells have been shown to promote functional recovery of peripheral nerve injuries in animal models.^17^,^35^

Cho et al^24^ demonstrated the use of neural-induced MSCs in peripheral nerve (facial nerve) regeneration after transection in an animal model. The advantages of bone marrow stromal cells are their apparent plasticity and ease of harvest. These MSCs can be harvested from long bones and have shown promise when used in combination with other proposed methods of nerve repair, including artificial conduits and acellular grafts. However, the ability of bone marrow stromal cells to produce a bona fide myelinating cell in vivo has been questioned.^36^

Amoh et al^28^ demonstrated the use of human hair follicle pluripotent stem cells in promoting regeneration of peripheral nerve injury. Induced pluripotent stem cells are pluripotent stem cells that have been produced from skin cells by either viral-, plasmid-, or transposon-mediated gene transfer. In addition to the risks of viral-mediated transfers, the other disadvantages of induced pluripotent stem cells include a possible malignant potential and inefficient production.

Amoh et al^28^ also discussed how embryonic stem cells (ESCs) have shown promise for pluripotency. In addition, Kubo et al^33^ demonstrated how ESC-derived motor neurons form neuromuscular junctions in vitro and enhance motor functional recovery in vivo. Although embryos are considered the best source of stem cells, ethical issues were the major challenge in the use of human ESCs (hESCs) for both research and clinical applications. Therefore, considerable interest focused on adult stem cells. However, most adult stem cells are relatively sparse and in indeterminate locations and growth states.^37^ Although Walsh and Midha^17^ reported a list of ongoing studies using stem cells from various sources for peripheral nerve repair, none of them use hESCs. Some studies have shown that hESCs^38^^-^41 are a potential source for cell therapy in regenerative neurology, although they did not specifically evaluate for peripheral nerve repair. The lift of the ban on using hESCs provided an opportunity to use them now for research and therapeutic applications. We initiated an effort to derive neural stem cells (NSCs) from hESCs for potential therapeutic application toward peripheral nerve regeneration.

## THE ROLE OF NEURAL STEM CELLS IN NERVE REGENERATION

*Neural stem cells* are defined as immature, uncommitted cells that are widely distributed in the embryonic, fetal, and adult nervous systems. NSCs can produce homologous new cells after nerve tissue injury and can continuously be transplanted. They have the ability of self-renewal and potentiality to differentiate into neurons upon delivery of appropriate signals.^42^ This plasticity of NSCs could potentially be used to promote neurogenesis following injury and disease. NSC transplantation has been shown to be a promising tool for restoring the nervous system in a variety of neurodegenerative disorders.^35^,^42^,^43^

Studies by Gu et al^43^ in 2010 demonstrated that NSCs transplanted into peripheral nerve can differentiate into neurons. They have shown that fetal NSCs transplanted into peripheral nerves could differentiate into neurons and form functional neuromuscular junctions with denervated muscle, which may be beneficial for the treatment of peripheral nerve injury.^43^

In the relatively restricted space of a peripheral nerve injury, continuously self-renewing NSCs can replenish the transplanted ones, and their multipotent differentiation capacity can induce differentiation into neurons and other neural cells. The self-renewal potential of NSCs makes them suitable for peripheral nerve transplantation. In addition, the transplanted NSCs can alter the local microenvironment, presumably by secreting neurotrophic factors that would promote axon regeneration.^44^

It has now been also confirmed that NSCs that are implanted into the nervous system can promote axonal regeneration to form SC-like peripheral myelin.^45^,^46^ Schwann cells not only form the main structure but also are the functioning cells of peripheral nerves. They also play an important role in nerve regeneration and functional recovery after peripheral nerve injury.^20^^-^23,47,48

Grafted NSCs have been shown to potentially replace cells lost after surgical procedures such as peripheral nerve rerouting in spinal cord injury, which affects significant motor and sensory function. Since spinal cord nerves have less regenerative potential than peripheral nerves, the grafted stem cells could improve the regenerative potential of peripheral nerves and thereby establish new neuronal connections in degenerative and traumatic diseases of the nervous system.^49^,^50^

In a study conducted by Guo et al^42^ in 2009, NSCs were implanted into collagen protein sponge containing growth factors to construct tissue-engineered artificial nerve, repairing 10-mm facial nerve defects. The regenerative nerve grew over anastomotic stoma of the distal end 12 weeks after operation. Compared with a nerve autograft group, the regenerative medullated nerve fibers, fiber diameter, myelin sheath thickness, and latency period and amplitude of neuromuscular action potentials had no significant differences. However, compared to a group without NSCs, significant differences were noted, indicating that NSCs indeed do play a certain promotive role in nerve regeneration.^42^

In this same study, immunohistochemical staining results showed that there was a large group of BrdU (5′-bromouracil, a marker) positive cells in bridge grafting and also present in 1 mm of the distal end of regenerative nerve. This demonstrated that not only can NSCs survive and migrate, but they have a high division growth in nerve-bridge grafting as well. At the same time, it was also found that the S100 marker, SC-like phenotype, was positive in partial positive BrdU cells. This led to speculation that the transplanted NSCs differentiated into SCs and formed a Büngner zonal structure to guide axon growth, secrete a variety of nerve growth factors, and express a variety of cell-adhesion molecules, which further facilitate nerve regeneration.^42^,^51^

## THE ROLE OF NO/cGMP SIGNALING IN NEURAL CELL DIFFERENTIATION

This review focuses on the role of nitric oxide (NO)/cyclic guanosine monophosphate (cGMP) signaling in hESC-derived neural cell differentiation, which allows us to further understanding the relationship of stage-specific stem cell markers with NO/cGMP signaling molecules during neural cell differentiation. Nitric oxide, a diffusible messenger of many forms of intercellular communication and intracellular signaling, regulates crucial physiological processes in the nervous system such as learning, memory, and neuronal survival and differentiation. Cyclic guanosine monophosphate is a cyclic nucleotide derived from guanosine triphosphate. Cyclic guanosine monophosphate acts as a second messenger much like cyclic adenosine monophosphate (cAMP), most notably by activating intracellular protein kinases in response to the binding of membrane-impermeable peptide hormones or NO to the external cell surface.^52^ Cyclic guanosine monophosphate synthesis is catalyzed by guanylate cyclase (GC), which converts GTP to cGMP. Soluble GC is typically activated by NO to stimulate cGMP synthesis.

NO regulates synapse formation and patterning, thus playing a role in embryonic and adult neurogenesis and development. Research efforts have shown that NO is a modulator of axon outgrowth and guidance, synaptic plasticity, neuronal precursor proliferation, and neuronal survival.^53^ NO plays a vital role in the self-renewal of NSCs. In addition, NO mediates communication between presynaptic and postsynaptic structures, which are required for normal sensorimotor function and development in adult mammals. NO also modulates the transmission of autonomic neural activity to target organs by actions within the spinal cord, ganglia, and neuromuscular junctions. The resultant complex interaction of NO with autonomic functions implies that pathophysiological changes in the synthesis and metabolism of NO may have direct consequences for neural control. It has been shown that NO regulates NSC (nestin+) proliferation and, possibly, differentiation into neurons. As NO is an important regulator of nervous system function, substantial changes in NO and cGMP synthesis may lead to nervous system degeneration. Regulation of NO and cGMP formation in the nervous system has been the subject of extensive studies for many years.^54^^-^56

## NO SIGNALING COMPONENTS

**Figure 1:**
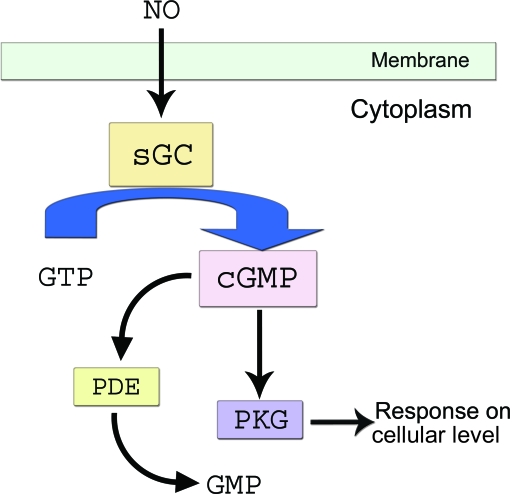
Nitric oxide signaling pathway. NO indicates nitric oxide; sGC, soluble guanylate cyclase; GTP, Guanine triphosphate; cGMP, cyclic Guanine monophosphate; PDE, phosphodiesterase; PKG, protein kinase G.

NO signaling is one of the most studied and significant signaling pathways (Fig 1) in many cells and tissues, including cardiac and neural systems. Soluble guanylyl cyclase (sGC) is a heme-containing, heterodimeric NO receptor that synthesizes cGMP. Soluble guanylyl cyclase consists of 2 subunits, α and β, which make up the active enzyme. The sGC α and β subunits exist in a 1:1 stoichiometry. Soluble guanylyl cyclase can be activated by NO, which can bind up to 400-fold.^56^ Recently, we have demonstrated the differential expression of NO signaling components including NO synthase (NOS-1, -2, -3), their receptors (sGC α1 and β1), and protein kinase G in murine ESCs, ESC-derived cardiomyocytes, and for the first time in hESCs.^56^ We have previously demonstrated the role of the NO–cGMP signaling pathway in differentiation of human and mouse ESCs into myocardial cells by regulating the expression of the NO receptor, sGC.^53^,^55^,^56^

## THE EXPRESSION PATTERNS OF sGC SUBUNITS IN hESC DIFFERENTIATION

We have reported in our earlier studies^56^ the differential expression of NO signaling components in differentiation of mouse ESCs^55^ and have shown differential expression of various genes in undifferentiated and differentiated murine stem cells. In another study, we examined the role of NO signaling in hESCs by looking at different subunits of sGC, proteins, and other markers in undifferentiated cells and during various stages of differentiation using hESCs (H-9). Our studies indicated that there was a significant increase in the expression of sGC α1, α2, and β1 during different stages of ESC differentiation at the mRNA and protein levels. These results clearly correlated with our earlier study^55^ for sGC α1 and β1 expression at the mRNA and protein levels in mouse ESCs. However, as the cells progressed toward differentiation, mRNA expression of sGC β2 declined slowly at the day-8 embryoid body stage and was completely undetectable at days 15 to 25 postdifferentiation. The β2 subunit that is predominantly expressed in the kidney has been shown to have activity in the absence of other subunits.^57^ In addition, upregulation of this subunit has been shown in gastric carcinoma tissues.^58^ Therefore, it is possible that this subunit may have some role in pluripotency of ESCs.

In addition to mRNA levels of different subunits, there was a concomitant increase in protein levels of sGC α1 and β1 during different stages of hESC differentiation to cardiomyocytes. Our previous^55^ report provided the evidence that ESC-derived cardiomyocytes express a functional sGC enzyme that can be activated upon NO stimulation to produce cGMP.

In summary, it was shown that there is a marked and time-dependent increase in the mRNA expression of both sGC subunits in differentiated cells relative to undifferentiated hESCs. On the basis of the data of our previous study with mouse ESCs and this report using hESCs, along with studies of other investigators with mouse ESCs, we believe that NO may play a significant role in the differentiation of stem cells.^56^

## CORRELATION OF hESC DIFFERENTIATION AND STAGE MARKERS WITH THE NO SIGNALING MOLECULE

**Figure 2:**
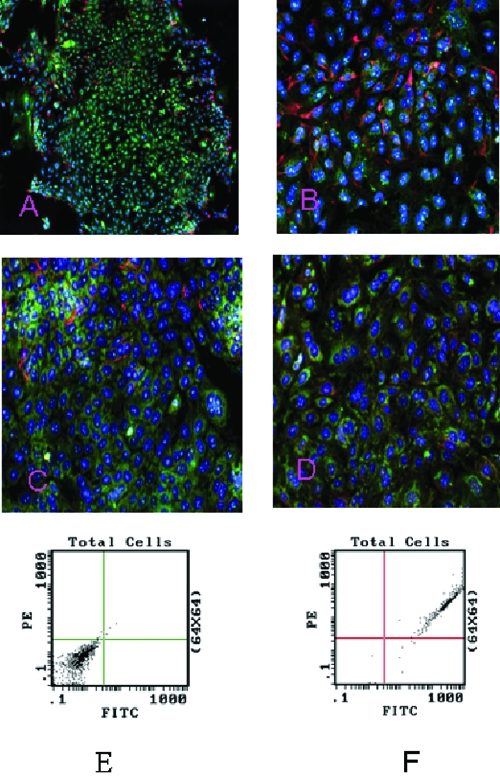
Immunofluorescence labeling of human embryonic stem cell (hESC)–derived neural stem cells (NSCs) showing coexpression of neural stem cell marker, nestin, and nitric oxide synthase (NOS)-1 in stage 1 hESC. Nestin-PE (*red*) and NOS-1 FITC (*green*) expression in stage 1 hESC-5x (A). Nestin and NOS-1 expression in hESC derived NSCs in differentiation medium-20x (B). Nestin and NOS-1 expression in hESCs treated with retinoic acid (10^−6^ M) (C). Combined treatment of retinoic acid (10^−6^ M) and 8-brom-cGMP (10^−5^ M) (D). Fluorescence activated cell sorter analysis of stage 1–differentiated hESCs for CD-133 and NOS-1 (E-F). Right panel (E) showing 97.5% of cells coexpressing CD-133 and NOS-1 (darker color). Left panel (F) was the result of negative control.

Although other researchers have demonstrated that hESC could be efficiently induced to differentiate into neural cells, we demonstrate the role of NO signaling molecules in hESC differentiation into neural cells for the first time. Figure 2A shows the confocal image of stage 1 hESCs coexpressing the neural precursor cell markers nestin-PE (red) and NOS-1 fluorescein isothiocyanate (FITC) (*green*). NOS-1–expressing cells accumulated in the center, while nestin-expressing cells diffused throughout the entire area. Cells co-staining for both the markers are present throughout the field (Fig 2B). Further study with fluorescence-activated cell sorting (FACS) analysis revealed that 97.5% of cells coexpressed CD-133 and NOS-1 as shown in Figures 2E and 2F. Coexpression of CD-133 and nestin also were detected in stage 1–differentiated hESCs (data not shown).

## EFFECT OF NO SIGNALING ON DIFFERENTIATION OF hESC TO NEURAL CELLS

We hypothesize that NO/cGMP signaling molecules regulate neural lineage commitment and govern neural precursor differentiation. The methodology and data presented later provide valuable insight into the effect of NO signaling on the differentiation of hESCs to neural cells.

To prepare the cell, H-9 cells (WA-09 or hESCs) were purchased from Wi Cell Institute (Madison, Wis). The cells were grown on 80% Dulbecco's Modified Eagle Medium with F12, 20% knockout serum replacer, L-glutamine (1 mmol/L), α-mercaptoethanol (0.1 mmol/L), and nonessential amino acids (1 mmol/L) supplemented with basic fibroblast growth factor (4 ng/mL). All of the reagents that were used for maintenance of the hESC were purchased from Invitrogen Corporation. H-9 cells were initially grown, and routinely maintained on a mitomycin C–treated mouse embryonic fibroblast (MEF) feeder layer, and subsequently cultured on Matrigel (BD Biosciences) for feeder-free culture with MEF-conditioned media supplemented with 4 ng/mL of basic fibroblast growth factor. The cells were routinely passed after every 5 to 6 days. To remove MEF, the cells were passed on to Matrigel and then used for differentiation for all the experiments.

Figure 2B shows the coexpression of nestin and NOS-1 in the differentiation medium of the hESC culture. FACS analysis indicated a 24.3% positive labeling of NOS-1. Adding retinoic acid (10^−6^ M) to the culture medium^59^ (Fig 2C) increased the percentage of NOS-1 positive cells to 33.9%. Combining retinoic acid (10^−6^ M)^55^ with 8-brom cGMP(10^−5^ M),^60^ the FACS analysis demonstrated a further increase of NOS-1 positive cells to 45.4% (Fig 2D). Our current results demonstrate a prodifferentiation potency of NOS-1, stimulated by retinoic acid with and without cGMP. Both retinoic acid and cGMP are known for their positive effects on hESC differentiation.

## CONCLUSION

Although significant progress has been made by us and others, the mechanism of NO/cGMP signaling in NSC differentiation and peripheral nerve regeneration has still not yet been completely defined. An important finding described in this article, however, is that NO/cGMP signaling contributes to neural precursors derived from hESC and enhances the differentiation of precursors toward functional neurons. Therefore, hESCs can potentially be used in repairing peripheral nerve injury by differentiating into functional neurons and regenerating injured peripheral nerves. This opens the door for therapeutic application toward healing and curing degenerative, traumatic, and other nervous system disorders. Further studies to elucidate more details of NO/cGMP signaling involvement in NSC differentiation and peripheral nerve regeneration are encouraged. Future clinical applications may include development of pharmacological protocols targeting the components of the NO/cGMP pathway (NOS, sGC, cGMP-dependent protein kinases, phosphodiesterases), as well as activation/inhibition to influence neural fate, neural precursor proliferation and differentiation, and peripheral nerve regeneration.

## Acknowledgment

We would like to thank Mr Aniket Mehta, MS, for his technical assistance in this article.
